# Exploration of the combined role of immune checkpoints and immune cells in the diagnosis and treatment of ankylosing spondylitis: a preliminary study immune checkpoints in ankylosing spondylitis

**DOI:** 10.1186/s13075-024-03341-6

**Published:** 2024-06-04

**Authors:** Feihong Huang, Zhiping Su, Yibin Huang, Yuxiang Huang, Chengyu Zhou, Sitan Feng, Xiong Qin, Xi Xie, Chong Liu, Chaojie Yu

**Affiliations:** 1 Department of Bone and Soft Tissue Surgery, Guangxi Medical University Cancer Hospital, Nanning, Guangxi Zhuang Autonomous Region 530021 China; 2 Guangxi Medical University, Nanning, Guangxi Zhuang Autonomous Region 530021 China; 3 Department of Radiation Oncology, Guangxi Medical University Cancer Hospital, Nanning, Guangxi Zhuang Autonomous Region 530021 China; 4 Spine and Osteopathy Ward, The First Affiliated Hospital of Guangxi Medical University, Nanning, Guangxi Zhuang Autonomous Region 530021 China; 5 Guangxi Key Laboratory of Regenerative Medicine, Orthopaedic Department, The First Affiliated Hospital of Guangxi Medical University, Nanning, Guangxi Zhuang Autonomous Region 530021 China

**Keywords:** Immune checkpoints, Ankylosing spondylitis, Immune cell, Drug sensitivity, Proteomic sequencing

## Abstract

**Objective:**

Immune checkpoints have emerged as promising therapeutic targets for autoimmune diseases. However, the specific roles of immune checkpoints in the pathophysiology of ankylosing spondylitis (AS) remain unclear.

**Methods:**

Hip ligament samples were obtained from two patient groups: those with AS and femoral head deformity, and those with femoral head necrosis but without AS, undergoing hip arthroplasty. Label-Free Quantification (LFQ) Protein Park Analysis was used to identify the protein composition of the ligaments. Peripheral blood samples of 104 AS patients from public database were used to validate the expression of key proteins. KEGG, GO, and GSVA were employed to explore potential pathways regulated by immune checkpoints in AS progression. xCell was used to calculate cell infiltration levels, LASSO regression was applied to select key cells, and the correlation between immune checkpoints and immune cells was analyzed. Drug sensitivity analysis was conducted to identify potential therapeutic drugs targeting immune checkpoints in AS. The expression of key genes was validated through immunohistochemistry (IHC).

**Results:**

HLA-DMB and HLA-DPA1 were downregulated in the ligaments of AS and this has been validated through peripheral blood datasets and IHC. Significant differences in expression were observed in CD8 + Tcm, CD8 + T cells, CD8 + Tem, osteoblasts, Th1 cells, and CD8 + naive T cells in AS. The infiltration levels of CD8 + Tcm and CD8 + naive T cells were significantly positively correlated with the expression levels of HLA-DMB and HLA-DPA1. Immune cell selection using LASSO regression showed good predictive ability for AS, with AUC values of 0.98, 0.81, and 0.75 for the three prediction models, respectively. Furthermore, this study found that HLA-DMB and HLA-DPA1 are involved in Th17 cell differentiation, and both Th17 cell differentiation and the NF-kappa B signaling pathway are activated in the AS group. Drug sensitivity analysis showed that AS patients are more sensitive to drugs such as doramapimod and GSK269962A.

**Conclusion:**

Immune checkpoints and immune cells could serve as avenues for exploring diagnostic and therapeutic strategies for AS.

**Supplementary Information:**

The online version contains supplementary material available at 10.1186/s13075-024-03341-6.

## Introduction

Ankylosing spondylitis (AS), a chronic inflammatory disease, is marked by immune system dysfunction, persistent inflammation, and joint stiffness [1]. The human leukocyte antigen class I molecule B27 (HLA-B27) is closely linked to AS. Upon exposure to specific triggers like infections and environmental factors, individuals with AS may experience a misdirected immune response, wherein the protein encoded by the HLA-B27 gene prompts the immune system to attack self-tissues, particularly in the spine and joints [2]. Current AS treatment focuses on reducing joint pain and inflammation with nonsteroidal anti-inflammatory drugs (NSAIDs) [3], but it doesn’t impede AS progression. As AS advances, stiffness and fusion of the spine and pelvic joints develop, significantly impacting daily activities [4]. While recent studies show promising progress with immune checkpoint inhibitors in cancer and autoimmune diseases [5], their correlation with AS is limited. This study aims to identify immune checkpoints in AS progression, analyze regulatory patterns of immune checkpoint genes (ICGs) in AS, and propose new treatment strategies.

ICGs encode surface proteins on immune cells, regulating cell activation and inhibition through interactions to maintain immune system balance [6]. Common immune checkpoint genes, including CTLA-4, PD-1, and PD-L1, negatively regulate immune responses by inhibiting activation signals or modifying immune cell functions [7]. Therapeutic approaches targeting these genes involve immune checkpoint inhibitors and agonists. Inhibitors, such as anti-CTLA-4 (e.g., ipilimumab) and anti-PD-1/PD-L1 antibodies (e.g., pembrolizumab, nivolumab, atezolizumab, durvalumab), enhance the immune system’s ability to combat tumor cells by blocking inhibitory signals [8–11]. Conversely, agonists stimulate excitatory signals, promoting immune cell activation and enhancing responses [12]. Recent research suggests an overly activated immune system in AS patients, with insufficient immune checkpoints to suppress autoimmune reactions [13]. This may contribute to immune cell attacks on body tissues, exacerbating AS progression.

AS, being intricately linked to the immune system, may have its pathogenesis regulated by immune checkpoints. In this study, gene and protein expression data from ligament and peripheral blood samples of AS patients were collected. Differential and intersection analyses with ICGs were conducted to identify closely associated ICGs. Subsequent investigations included exploring pathways and immune cells regulated by these ICGs, along with drug sensitivity analysis. Through a comprehensive analysis centered on AS-associated ICGs, this study aims to offer novel insights for AS treatment.

## Materials and methods

### Sample collection

Hip ligament samples were collected from patients undergoing hip arthroplasty at the Affiliated Hospital of Guangxi Medical University between January 2018 and September 2019. The control group included patients with femoral head necrosis (*n* = 6), while the experimental group comprised patients with AS combined with femoral head deformity (*n* = 6). Exclusion criteria involved rheumatoid arthritis, systemic lupus erythematosus, and tumors. The study received approval from the Ethics Committee of the Affiliated Hospital of Guangxi Medical University, and informed consent was obtained from all participants. Three in each group were used for proteomic studies (3AS v 3Control) and three in each group for immunohistochemical analysis (3AS v 3Control).

### Proteomics analysis

**Fig. 1:**
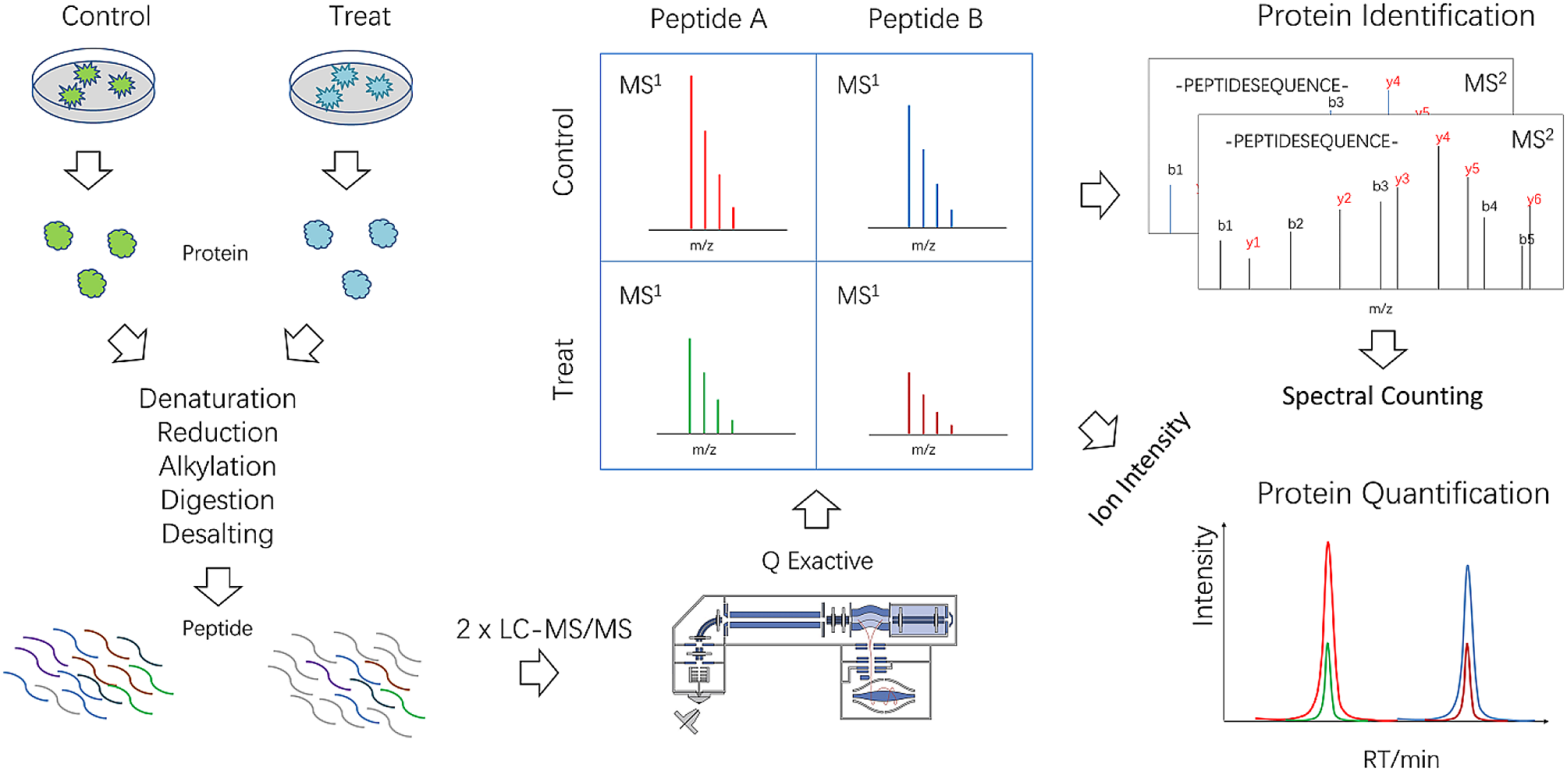
The flowchart of label-free quantification (LFQ) protein park analysis

Label-Free Quantification (LFQ) Protein Park Analysis was conducted on hip ligament samples. Relative protein expression levels were determined through the quantification of peptide identifications and peak areas in Mass Spectrometry Level 1 (MS1). Experimental steps encompassed sample processing, protein quantification, acetone precipitation, protein solubilization, and reduction. Desalted peptides were separated using the nano-UPLC system EASY-nLC1200 and detected with an online mass spectrometer (QExactive). Raw files underwent processing via MaxQuant (version 1.6.10), utilizing the protein database from the UNIPROT database (Uniprot_human_2018_10). The experimental workflow is depicted in Fig. 1.

### Identification of differentially expressed genes and proteins associated with immune checkpoint

From a literature review, we acquired a set of 79 ICGs [14] (supplementary materials1). Employing the STRING database (https://cn.string-db.org/), Protein-Protein Interaction (PPI) analysis on these 79 ICGs were conducted, and the results were visualized using Cytoscape_v3.10.0.

In the NCBI GEO database (https://www.ncbi.nlm.nih.gov/geo/), searches were performed using keywords such as Ankylosing Spondylitis, Spondyloarthritis, and SpA to gather gene expression matrices. Subsequent differential expression analyses for genes and proteins were carried out using the R package “limma.” Given that the mRNA expression levels in peripheral blood are lower while protein sequencing expression levels are higher, to precisely capture potentially biologically significant minor differential genes, we set a 1.1-fold difference threshold for peripheral blood mRNA data and a 2.0-fold difference threshold for protein sequencing data. Moreover, for the key genes identified, t-test was employed for a double verification to ensure that their differences are statistically significant.

### Identification of disease-associated module genes using weighted gene co-expression network analysis (WGCNA)

The primary objective of WGCNA was to convert associations between numerous genes and phenotypes into associations between a concise number of gene sets and phenotypes by constructing gene co-expression networks [15]. The datasets GSE25101 and GSE73754, comprising a total of 104 samples and 12,584 genes, were utilized. Using gene expression profiles, we calculated the Median Absolute Deviation (MAD) for each gene in the two datasets and excluded the bottom 50% of genes with the smallest MAD. The “goodSamplesGenes” function from the R package WGCNA was then employed to eliminate outlier genes and samples. Subsequently, a scale-free co-expression network was constructed using WGCNA, grouping individuals based on the presence of AS. The minimum module gene count was set to 66, with a sensitivity of 3 and a module merge threshold of 0.25.

### Kyoto encyclopedia of genes and genomes (KEGG) and gene ontology (GO) enrichment analysis

The latest gene annotations for KEGG Pathway were acquired from the KEGG rest API (https://www.kegg.jp/kegg/rest/keggapi.html). Subsequently, gene annotations for Gene Ontology (GO) were retrieved from the R package org.Hs.eg.db (version 3.1.0). Enrichment analysis was then conducted using the R package clusterProfiler (version 3.14.3). Genes were mapped to the background set using their respective gene annotations. The enrichment analysis was performed with a minimum gene set of 5 and a maximum gene set of 5000. Statistically significant results were defined by P values less than 0.05.

### Gene set variation analysis (GSVA)

GSVA analysis, short for Gene Set Variation Analysis, is an algorithm employed in Gene Set Enrichment Analysis (GSEA) to evaluate changes in the activity of gene sets related to pathways/functions [16]. In our study, we conducted KEGG enrichment analysis and intersection analysis to identify 12 significantly enriched common pathways. Genes enriched in these pathways were defined as predefined gene sets. Utilizing the R package GSVA (DOI:10.18129/B9.bioc.GSVA, version 1.40.1), we computed the enrichment scores for each sample in these gene sets, setting a minimum gene set size of 5 and a maximum gene set size of 5000. The resulting output was the enrichment score matrix.

### Calculating the levels of cell infiltration

xCell is a gene signature-based method capable of inferring 64 immune and stromal cell types [17]. For immune infiltration analysis, we utilized the Immuno-Oncology Biological Research (IOBR) tool, which integrates multiple algorithms [18]. Specifically, we employed the xCell algorithm (http://xCell.ucsf.edu/) through the R package IOBR to calculate infiltration scores for 64 cell types in peripheral blood samples in our study.

### Least absolute shrinkage and selection operator (LASSO) regression for screening key cells

To identify key cell types associated with the disease, the R package “glmnet” was employed. Integration of disease status and cell infiltration scores was performed, followed by regression analysis using the lasso-cox method. Subsequently, we obtained the risk scores for the model and conducted ROC analysis using the R package “pROC” (version 1.17.0.1).

### External validation of the diagnostic model

External validation of the diagnostic model was conducted using datasets GSE134290 (3AS v 3Control) and GSE11886 (9AS v 8 Control) from the NCBI GEO database. Both the gene-based diagnostic model and the model integrating genes and immune cells were applied to calculate the risk score for each sample using gene expression matrices. ROC analysis was performed using the R package “pROC” (version 1.17.0.1).

### Correlation analysis between immune checkpoint genes and immune cells

To examine the correlation between ICGs and immune cells associated with AS, we integrated gene expression data and cell infiltration scores. Subsequently, correlation analysis was conducted using both the Pearson and Spearman methods.

### Drug sensitivity analysis

Motivated by the application of immunomodulators, immunosuppressants, and biologics in the treatment of autoimmune diseases, we conducted a drug sensitivity analysis. The R package “oncoPredict” was employed for predicting drug sensitivity based on gene expression matrices and identifying drug-specific biomarkers [19]. Specifically, we categorized the samples into disease and control groups. Subsequently, we inputted the gene expression matrix and drug information from the drug sensitivity database. Finally, using the R package “oncoPredict,” we performed drug sensitivity analysis on the selected feature genes, where the drug’s 50% inhibitory concentration (IC50) was used to represent drug sensitivity, with a smaller IC50 indicating higher sensitivity.

### Immunohistochemistry

The experimental group included hip joint ligament tissues from patients with AS combined with femoral head deformity (*n* = 3), while the control group comprised hip joint ligament tissues from patients with femoral head necrosis (*n* = 3). Immunohistochemistry (IHC) was utilized to compare the expression differences of HLA-DMB and HLA-DPA1 between the experimental and control groups. Tissues were sectioned into slices with a thickness of 4–6 micrometers, and following experimental procedures such as baking, deparaffinization, hydration, and antigen retrieval, fully stained immunohistochemical sections were obtained from paraffin-embedded slices. Observations and image acquisition were conducted under a microscope. ImageJ software was employed to convert the images into 8-bit grayscale pictures, and the 8-bit grayscale images were then converted into optical density (OD) values. The integral optical density (IOD) and positive area were calculated. By dividing the IOD value by the positive area of the target protein, the Average Optical Density (AOD) was obtained. AOD reflects the concentration of the target substance per unit area, with a higher AOD value indicating a relatively higher expression level of the target protein. Statistical analysis was performed using IBM SPSS Statistics 22.0.

## Results

### Identification of differentially expressed genes and proteins associated with immune checkpoints

**Fig. 2:**
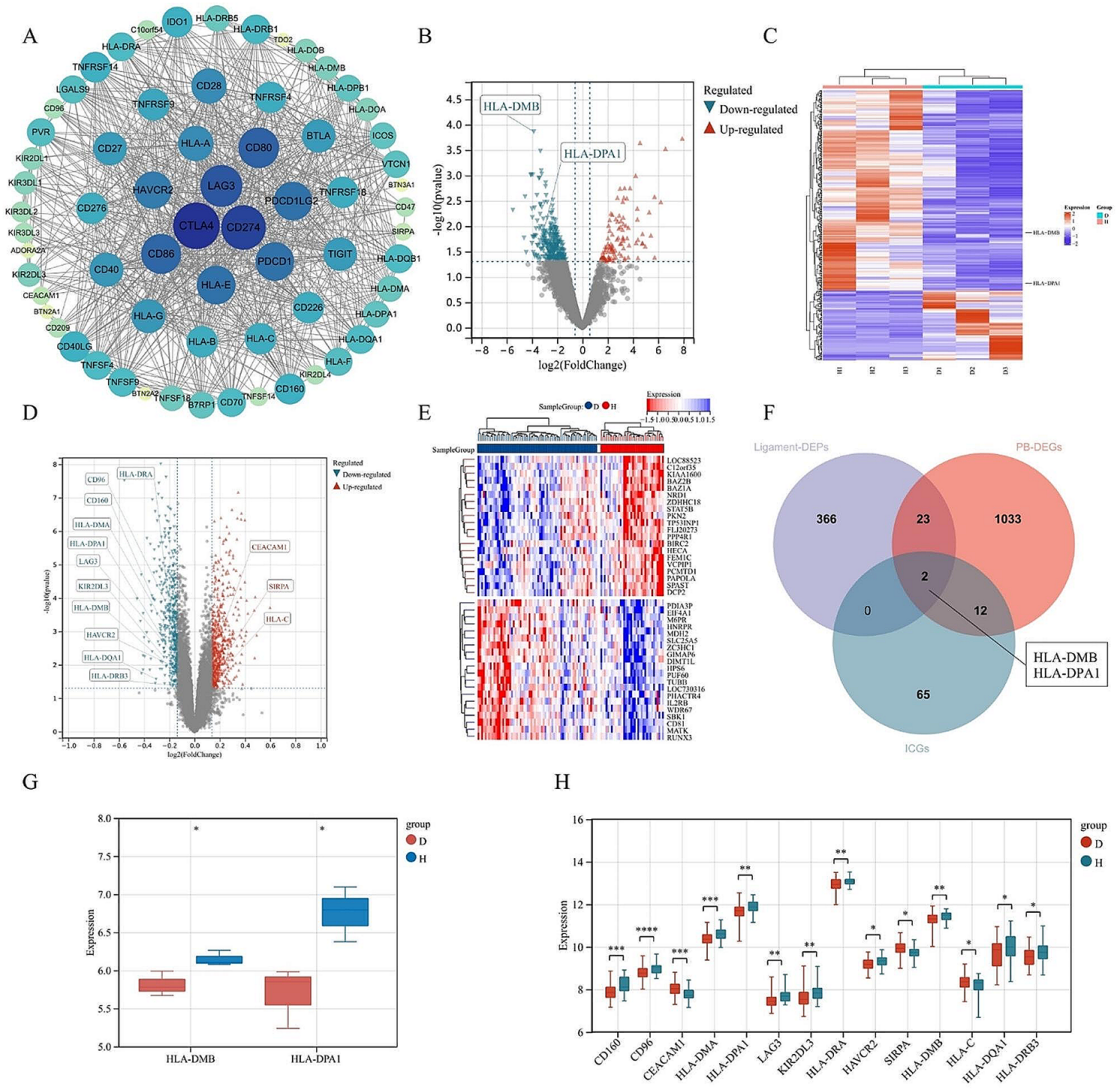
Selection of immune checkpoint genes (ICGs) and immune checkpoint proteins (ICPs) associated with Ankylosing Spondylitis (AS): (**A**) Protein-protein interaction (PPI) network of 79 ICGs: Each node represents a protein expressed by the corresponding gene, and the connections between nodes indicate protein-protein interactions. Larger nodes closer to the center indicate a higher number of associated proteins. (**B**) Volcano plot of protein expression differences. (**C**) Heatmap of protein expression differences, with expression > 0 indicating upregulation and expression < 0 indicating downregulation (D: disease group, H: healthy control group). (**D**) Volcano plot of gene expression differences. (**E**) Heatmap of gene expression differences, with expression > 0 indicating upregulation and expression < 0 indicating downregulation (D: disease group, H: healthy control group). (**F**) Intersection analysis of differentially expressed proteins (DEPs), ICGs, and differentially expressed genes (DEGs). (**G**) Expression levels of DEPs. (**H**) Expression levels of DEGs. (* indicates *p* < 0.05, ** indicates *p* < 0)

A protein-protein interaction (PPI) network of 79 ICGs demonstrated that all ICGs can be translated into their corresponding proteins, displaying universal connections among each other (Fig. 2A). LFQ protein park analysis identified a total of 3,489 proteins, with 391 being differentially expressed proteins (DEPs) (supplementary materials 2). Among the DEPs, 101 proteins were significantly upregulated, and 290 proteins were significantly downregulated (Fig. 2B, C).

Peripheral blood (PB) samples GSE25101 and GSE73754 were retrieved from the GEO database. Following batch correction, an mRNA expression matrix of 104 samples was obtained (Supplementary Material 3). Differential analysis identified 1,070 differentially expressed genes (DEGs) (Supplementary Material 4), including 611 genes significantly upregulated and 459 genes significantly downregulated (Fig. 2D, E).

By intersecting the ICGs with differentially expressed genes in peripheral blood (PB-DEGs), 14 differentially expressed immune checkpoint genes (DEICGs) were obtained (Fig. 2F), and their expression levels were validated through t-tests (Fig. 2H). Introducing DEPs into the intersection analysis revealed consistent downregulation of HLA-DMB and HLA-DPA1 at both the mRNA and protein levels (Fig. 2G).

### Identifies disease associated gene modules by WGCNA

**Fig. 3:**
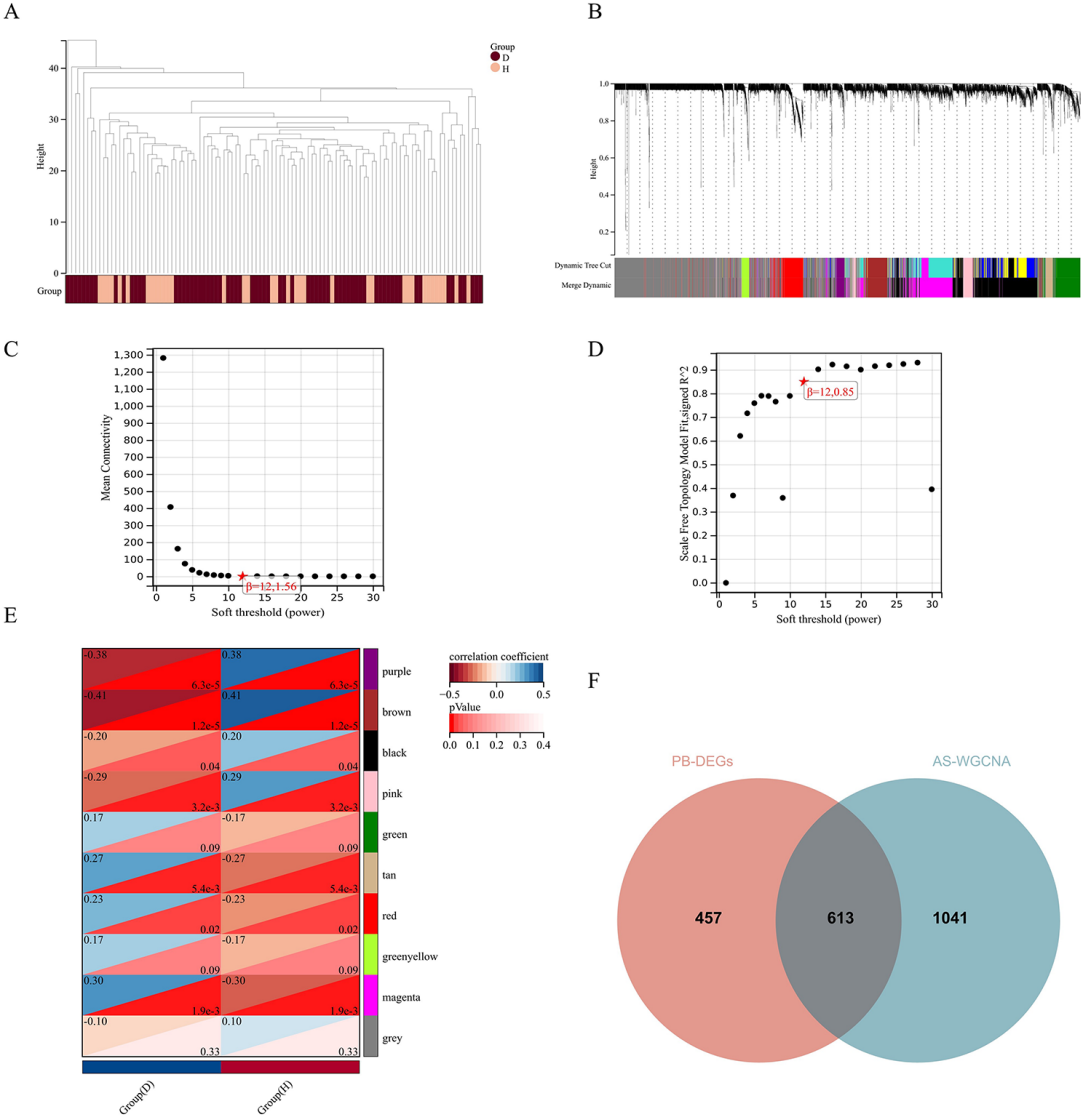
Weighted Gene Co-expression Network Analysis (WGCNA) for selecting AS-related gene modules: (**A**) Sample clustering (D: Disease group, H: Healthy control group). (**B**) Gene clustering. (**C**) Average connectivity curve. (**D**) Scale independence curve. (**E**) Gene modules (D: Disease group, H: Healthy control group). (**F**) Intersection analysis for selecting AS-related DEGs

WGCNA organizes genes into distinct modules, each comprising genes sharing similar expression patterns (Fig. 3A, B). The average connectivity curve and scale independence curve establish the ideal connectivity threshold and module size for WGCNA modules, regulated by the soft-thresholding parameter β (Fig. 3C, D). WGCNA identified a total of 10 modules, with the purple, brown, pink, tan, and magenta modules exhibiting significant statistical significance (*p* < 0.01) (Fig. 3E). We integrated the genes within these 5 modules to establish a gene set associated with AS, termed AS-WGCNA, comprising a total of 1654 genes. Through the intersection of AS-WGCNA with DEGs, 613 AS-related differentially expressed genes (AS-DEGs) were identified (Fig. 3F).

### The regulatory mechanism of immune checkpoint genes (ICGs) in AS

**Fig. 4:**
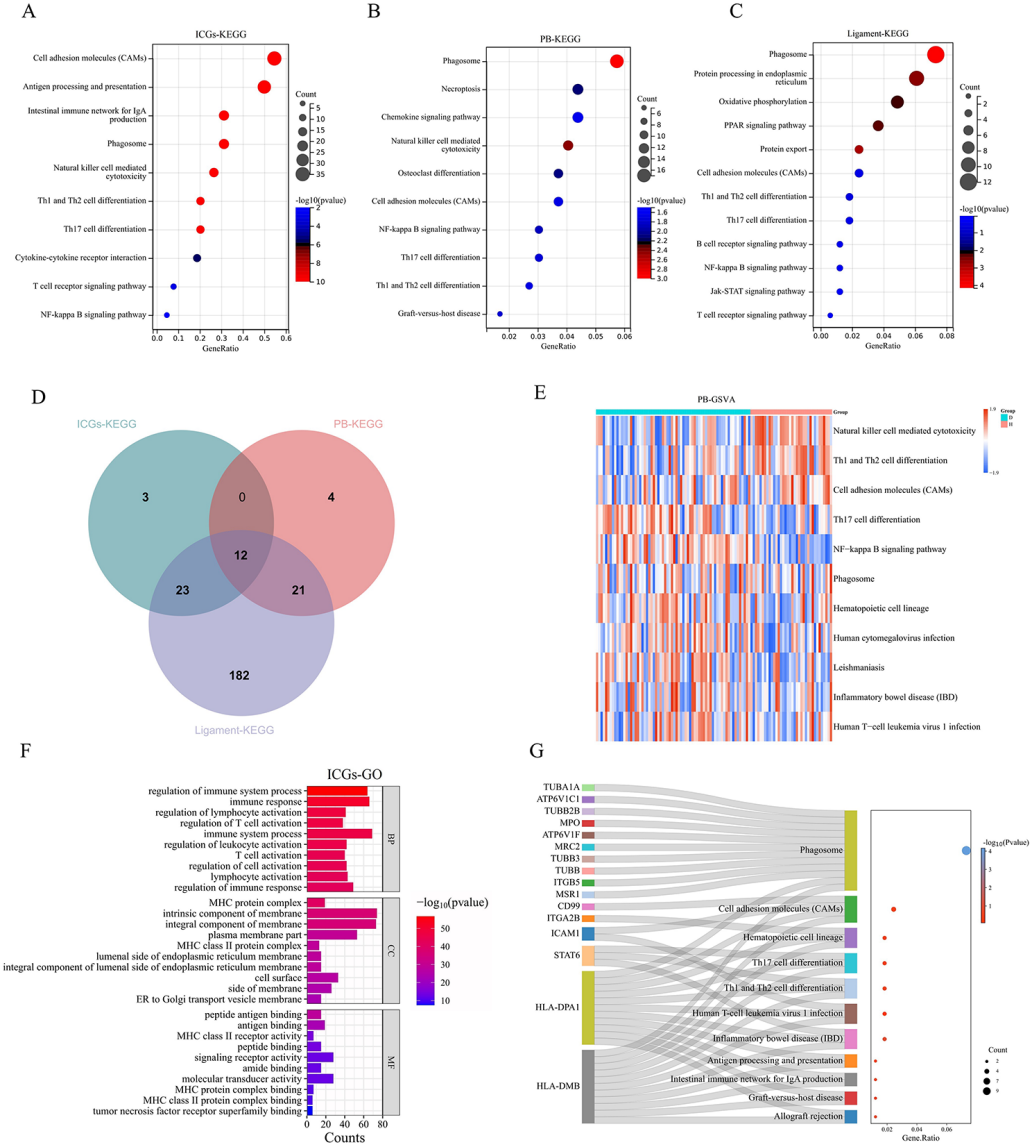
Pathways regulated by ICGs in AS: (**A**) KEGG pathway enrichment analysis of 79 ICGs. (**B**) KEGG pathway enrichment analysis of 613 AS-DEGs. (**C**) KEGG pathway enrichment analysis of 391 DEPs. (**D**) Intersection of enriched KEGG pathways among ICGs, AS-DEGs, and DEPs.(**E**) GSVA analysis of the common pathway gene set(Red indicates GSVA scores > 0, while blue indicates GSVA scores < 0.). (**F**) GO analysis of ICGs (BP: Biological Process, CC: Cellular Component, MF: Molecular Function). (**G**) Pathways regulated by HLA-DPA1 and HLA-DMB

KEGG analysis identified potential pathways regulated by ICGs, AS-DEGs, and DEPs in AS (Fig. 4A, B, C). Intersection analysis revealed 12 key common pathways, with 11 meeting the criteria for GSVA analysis due to having at least 5 enriched genes. GSVA analysis indicated significant activation of pathways like Th17 cell differentiation and the NF-kappa B signaling pathway, with inhibition observed in pathways such as Th1 and Th2 cell differentiation, and Natural killer cell-mediated cytotoxicity (Fig. 4D, E). GO analysis highlighted the association of ICGs with immune processes, T cells, and MHC complexes (Fig. 4F). The Sankey bubble plot demonstrated the involvement of HLA-DMB and HLA-DPA1 in regulating shared pathways like Th1 and Th2 cell differentiation, antigen processing and presentation, and Th17 cell differentiation (Fig. 4G).

### xCELL calculated the level of cellular infiltration

**Fig. 5:**
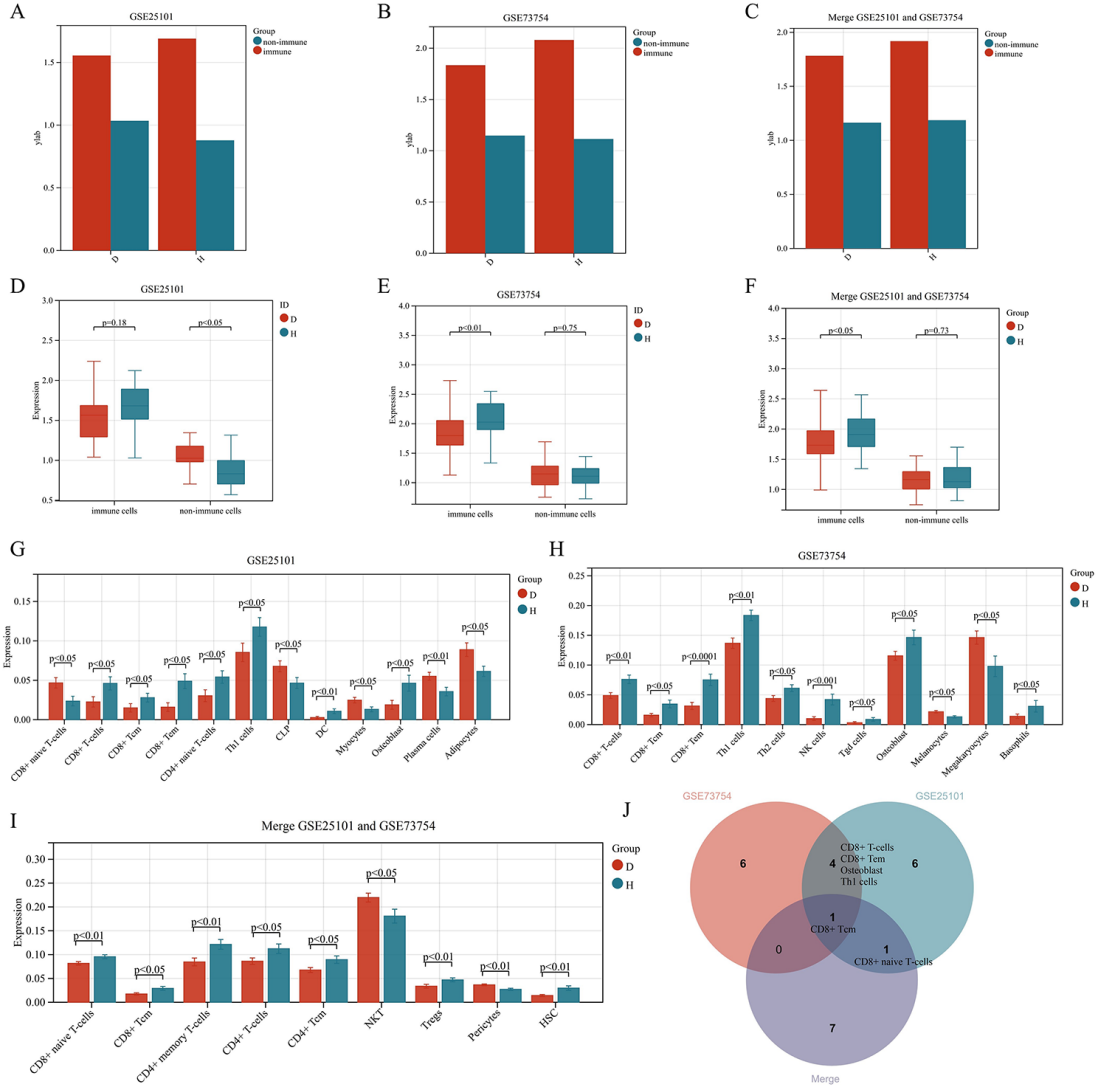
Analysis of cell infiltration levels in peripheral blood of AS patients using xCELL: (**A**, **B**, **C**) Cell composition of the peripheral blood dataset. (**D**, **E**, **F**) Differences in cell infiltration levels between the disease group and the control group. (**G**, **H**, **I**) Bar plots of significantly different infiltrating cells. (**J**) Intersection analysis of significantly different infiltrating cells

To identify key cells influencing the progression of AS, xCELL analyses and LASSO regression analyses were conducted on both pre-merged and post-merged samples. xCELL calculated the cell composition in PB samples, indicating a higher proportion of immune cells (Fig. 5A, B, C). As the sample size expanded from 36 cases to the merged 104 cases, the infiltration level of immune cells exhibited a significant decrease in the AS group (Fig. 5D, E, F). T-tests verified the differences in 64 cell infiltration scores between groups, revealing 12 significantly different cells in GSE25101 (Fig. 5G), 11 in GSE73754 (Fig. 5H), and 9 in the merged GSE25101 and GSE73754 datasets (Fig. 5I). Finally, through intersection analysis, key cells, including CD8 + Tcm, CD8 + T-cells, CD8 + Tem, Osteoblast, Th1 cells, and CD8 + naive T-cells, were identified (Fig. 5J).

### LASSO regression was employed to screen key cells

**Fig. 6:**
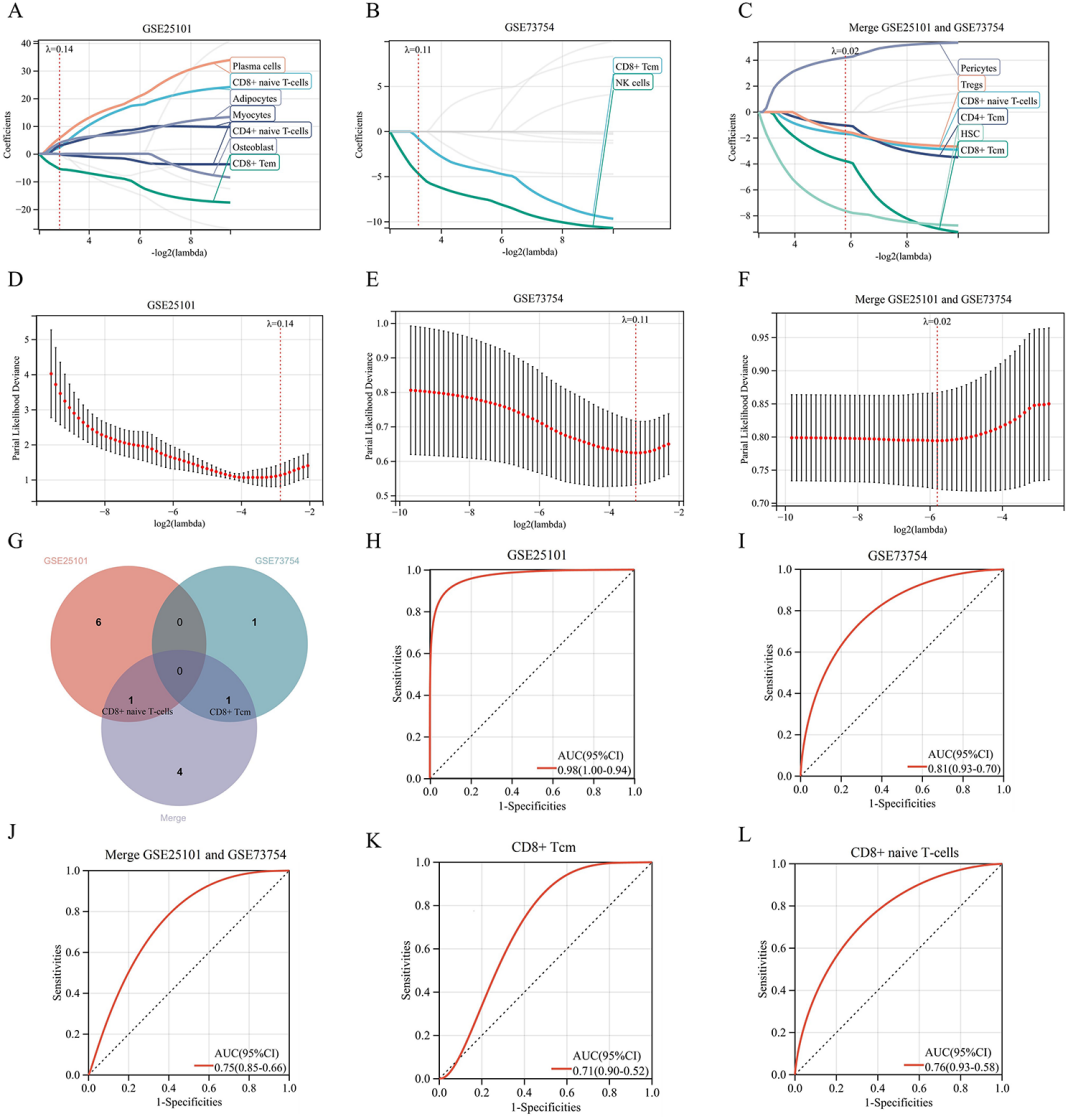
The selection of cells most relevant to AS from significantly different infiltrating cells: (**A**, **B**, **C**) LASSO regression for selecting cells most relevant to AS. (**D**, **E**, **F**) Selection of model parameters and cross-validation. (**G**) Intersection analysis. (**H**, **I**, **J**) ROC curves of AS diagnostic models based on immune cells. (**K**) ROC curve of CD8 + Tcm for predicting AS incidence. (**L**) ROC curve of CD8 + naive T-cells for predicting AS incidence

By conducting regression analysis on cells with significant differences in cell infiltration scores, LASSO regression was applied. For GSE25101, a Lambda value of 0.1386 was set, resulting in the selection of 7 cells out of 12 significantly different cells (Fig. 6A). For GSE73754, a Lambda value of 0.1058 was set, leading to the identification of 2 cells out of 11 significantly different cells (Fig. 6B). For the merged GSE25101 and GSE73754 dataset, a Lambda value of 0.0179 was set, and 6 cells were selected from 9 significantly different cells (Fig. 6C).To achieve the best model performance, the optimal λ value was determined through cross-validation (Fig. 6D-F).Through the intersection of the selected cells, CD8 + Tcm and CD8 + naive T-cells were identified (Fig. 6G).

In the diagnostic model constructed using the selected cells, GSE25101 had an AUC value of 0.98 (Fig. 6H), GSE73754 had an AUC value of 0.81 (Fig. 6I), and the merged GSE25101 and GSE73754 had an AUC value of 0.75 (Fig. 6J). The AUC value for CD8 + Tcm in predicting the disease was 0.71 (Fig. 6K), while for CD8 + naive T-cells, it was 0.76 (Fig. 6L). These results suggested that the selected cells may play an important role in the progression of AS. The formulas for the disease diagnostic models are following:

1. GSE25101: Risk Score = 2.2380CD8 + naive T-cells − 5.4756CD8 + Tem − 0.1179CD4 + naive T-cells + 2.9378Myocytes − 0.0960Osteoblast + 5.6258Plasma cells + 3.8903*Adipocytes.
2. GSE73754: Risk Score = -0.7776CD8 + Tcm − 4.6661NK cells.
3. Merge GSEGSE25101 and GSE73754: Risk Score = -1.6781CD8 + naive T cells − 3.7772CD8 + Tcm − 1.0301CD4 + Tcm − 1.5062Tregs + 4.2083Pericytes − 7.6209HSC.

(rounded to four decimal places)

### Diagnostic model constructed by combining genes and immune cells

**Fig. 7:**
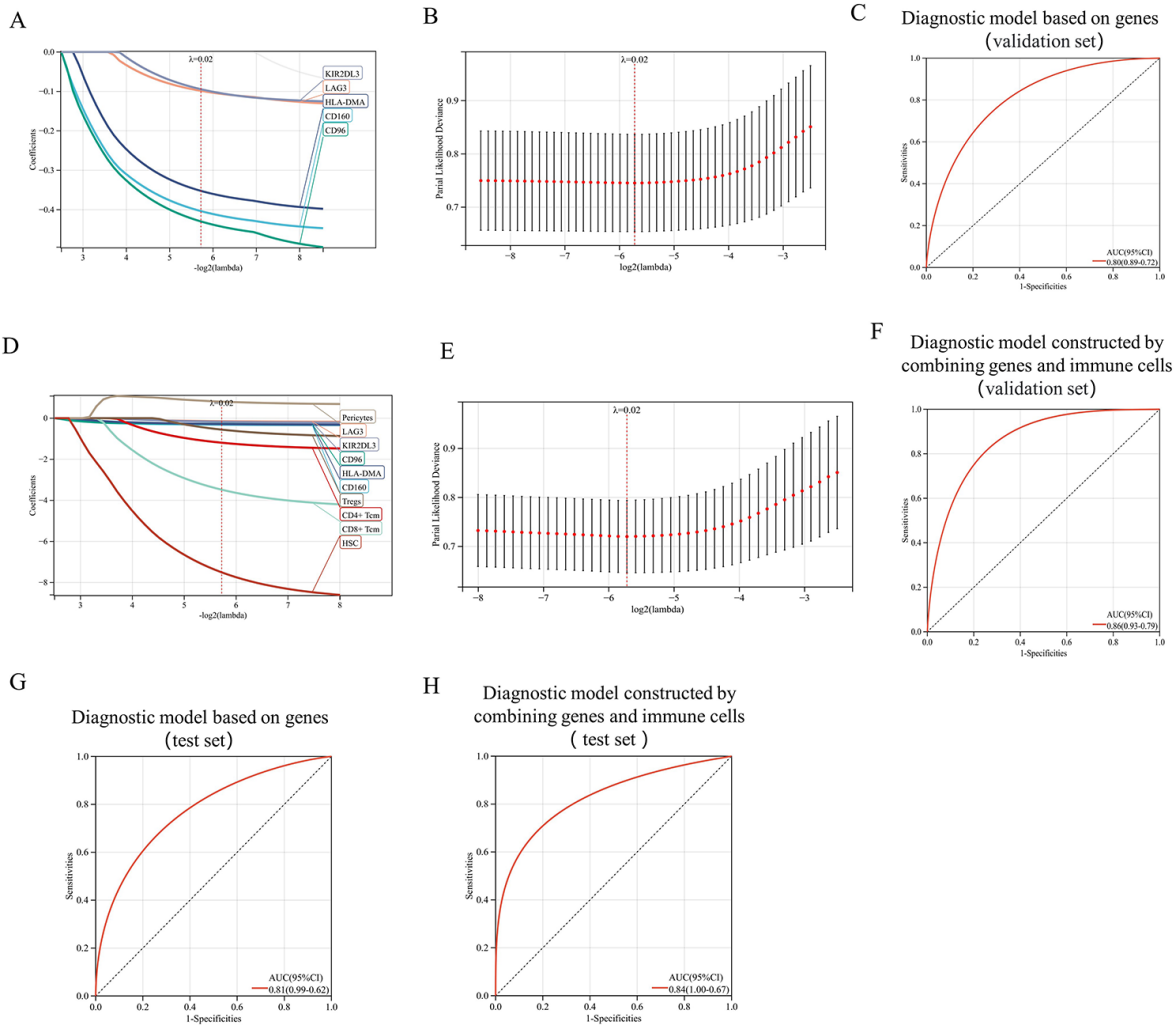
Uniting immune checkpoints genes(ICGs) and immune cells to construct a diagnostic model for ankylosing spondylitis(AS): (**A**, **B**) LASSO algorithm selection of ICGs contributing to AS diagnosis. (**C**)The ROC curve of validation set based on gene diagnostic model. (**D**, **E**) Development of an AS diagnostic model by combining ICGs and immune cells. (**F**) The ROC curve of validation set based on genes and immune cells diagnostic model.(**G**)The ROC curve of test set based on gene diagnostic model. (**H**)The ROC curve of test set based on genes and immune cells diagnostic model

**Fig. 8:**
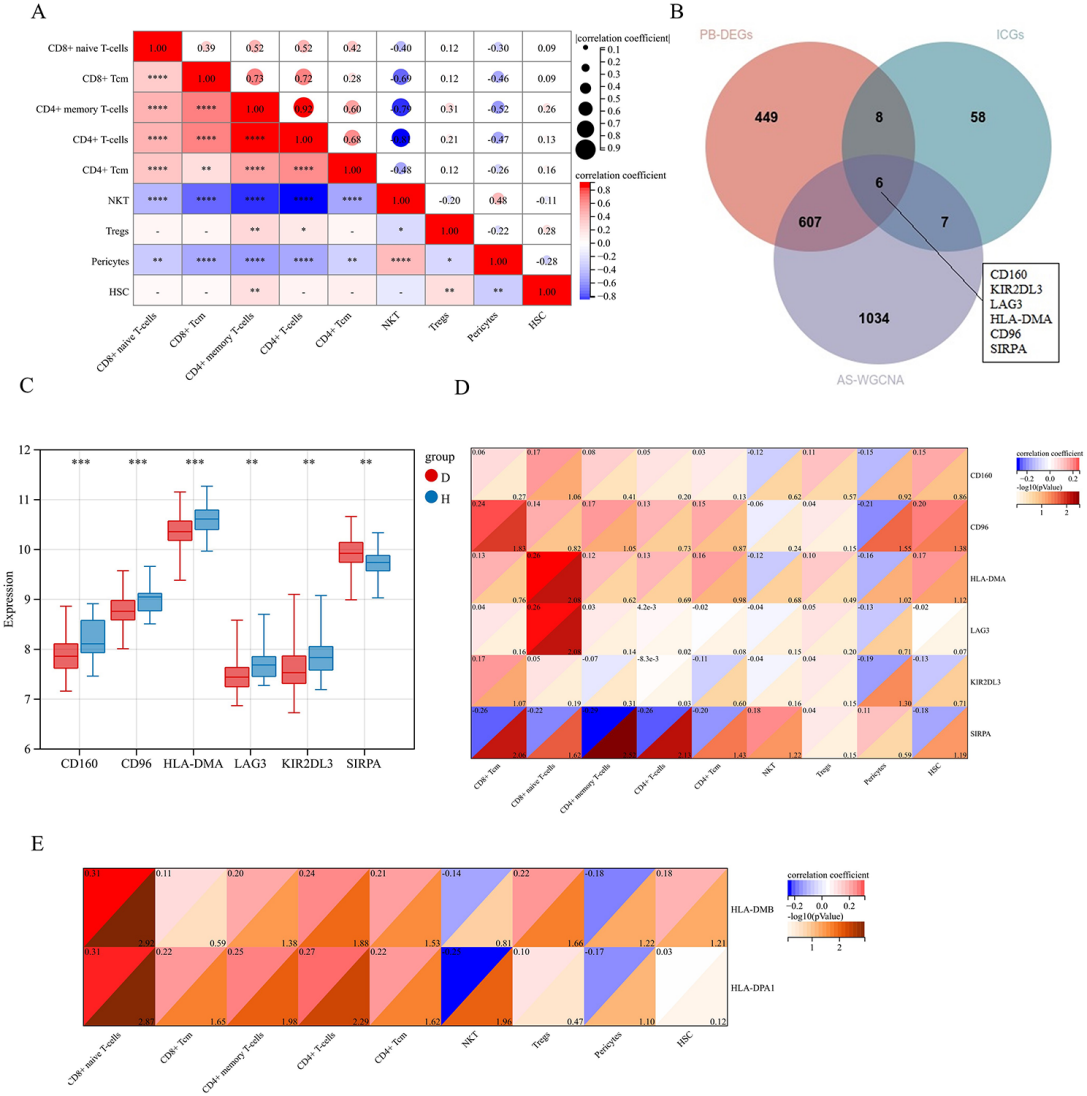
Correlation analysis between DEICGs and significantly different infiltrating cells: (**A**) Correlation analysis between 9 different cells from the merged datasets GSE25101 and GSE73754. (**B**) Further selection of key ICGs regulating AS. (**C**) The expression level of 6 key ICGs(D: disease; H:healthy control). (**D**)Correlation analysis between 6 key ICGs and 9 significantly different infiltrating cells. (**E**) Correlation analysis between DEPs and 9 significantly different infiltrating cells

The intersection of WGCNA, ICG, and PB-DEG resulted in six genes (Fig. 8A). LASSO regression analysis based on these six genes revealed that five of them hold diagnostic significance for the disease (Fig. 7A, B). Combining the six immune cell types from the merged GSE25101 and GSE73754 datasets (Fig. 6C) with the five genes demonstrating diagnostic value for AS, a novel diagnostic model for AS was constructed (Fig. 7D, E).

Merged datasets GSE25101 and GSE73754 served as validation sets for the diagnostic model. For gene-based disease diagnosis, the AUC value of the validation set was 0.80 (Fig. 7C), which increased to 0.86 when genes and immune cells were combined (Fig. 7F). Similarly, merged datasets GSE134290 and GSE11886 acted as test sets for diagnostic models. The gene-based diagnosis yielded an AUC value of 0.81(Fig. 7G), rising to 0.84 with the inclusion of immune cells (Fig. 7H). These results indicate excellent diagnostic efficiency, suggesting that combining immune checkpoints and cells enhances AS diagnosis efficacy.

The formulas for the disease diagnostic models are following:

(1) Diagnostic model based on genes:

Risk Score = -0.4035*CD160-0.4297*CD96-0.3522*HLA-DMA-0.0986*LAG3-0.0944*KIR2DL3.

(2) Diagnostic model constructed by combining genes and immune cells:

Risk Score = -0.3197*CD160-0.2770*CD96-0.2862*HLA-DMA-0.1461*LAG3-0.1750*KIR2DL3-3.4819*CD8+.

Tcm-1.1951*CD4 + Tcm-0.5581*Tregs + 0.8050*Pericytes-7.5060*HSC.

(rounded to four decimal places)

### Correlation analysis between ICGs and immune cells

A correlation analysis using the gene expression matrix and cell infiltration scores of 9 different cell types from the merged datasets GSE25101 and GSE73754 revealed a significant positive correlation between the infiltration levels of CD8 + Tcm and CD8 + naive T-cells (Fig. 8A).

To identify ICGs playing a regulatory role in the progression of AS, we further intersected the ICGs, AS-WGCNA, and PB-DEGs. Eventually, six key genes were obtained: CD160, CD96, HLA-DMA, LAG3, KIR2DL3, and SIRPA (Fig. 8B). In the disease group, CD160, CD96, HLA-DMA, LAG3, and KIR2DL3 were significantly downregulated, while SIRPA was significantly upregulated (Fig. 8C).

SIRPA showed a significant negative correlation with CD8 + Tcm, CD8 + naive T-cells, CD4 + memory T-cells, CD4 + T-cells, and CD4 + Tcm (Fig. 8D). CD96 showed a significant positive correlation with CD8 + Tcm, while HLA-DMA and LAG3 showed a significant positive correlation with CD8 + naive T-cells (Fig. 8D). HLA-DPA1 exhibited a significant positive correlation with CD8 + Tcm and CD8 + naive T-cells, whereas HLA-DMB showed a significant positive correlation with CD8 + naive T-cells (Fig. 8E). These findings suggested that CD8 + Tcm and CD8 + naive T-cells may be regulated by these genes.

#### Drug sensitivity analysis

We conducted drug sensitivity analysis targeting six genes and two proteins, including CD160, CD96, HLA-DMA, LAG3, KIR2DL3, SIRPA, HLA-DPA1, and HLA-DMB. The efficacy of 198 drugs targeting these genes/proteins was analyzed. The results showed that six drugs achieved IC50 at lower concentrations (Supplementary Fig. 1A-F), suggesting higher sensitivity of AS patients to these six drugs.

Correlation analysis between drug IC50 and gene expression levels revealed a significant positive correlation between the IC50 of doramapimod and downregulated genes/proteins HLA-DPA1, HLA-DMB, HLA-DMA, and KIR2DL3 (Supplementary Fig. 2A-D). The IC50 of GSK269962A was significantly positively correlated with downregulated genes/proteins HLA-DPA1, HLA-DMB, HLA-DMA, CD160, CD96, and LAG3 (Supplementary Fig. 2E-J) and significantly negatively correlated with upregulated gene SIRPA (Supplementary Fig. 2K). The correlation coefficient between doramapimod and HLA-DPA1 reached 0.96 (Supplementary Fig. 2A), while the correlation coefficient between GSK269962A and SIRPA reached − 0.98 (Supplementary Fig. 2K).

### Quantitative analysis for immunohistochemistry

**Fig. 9:**
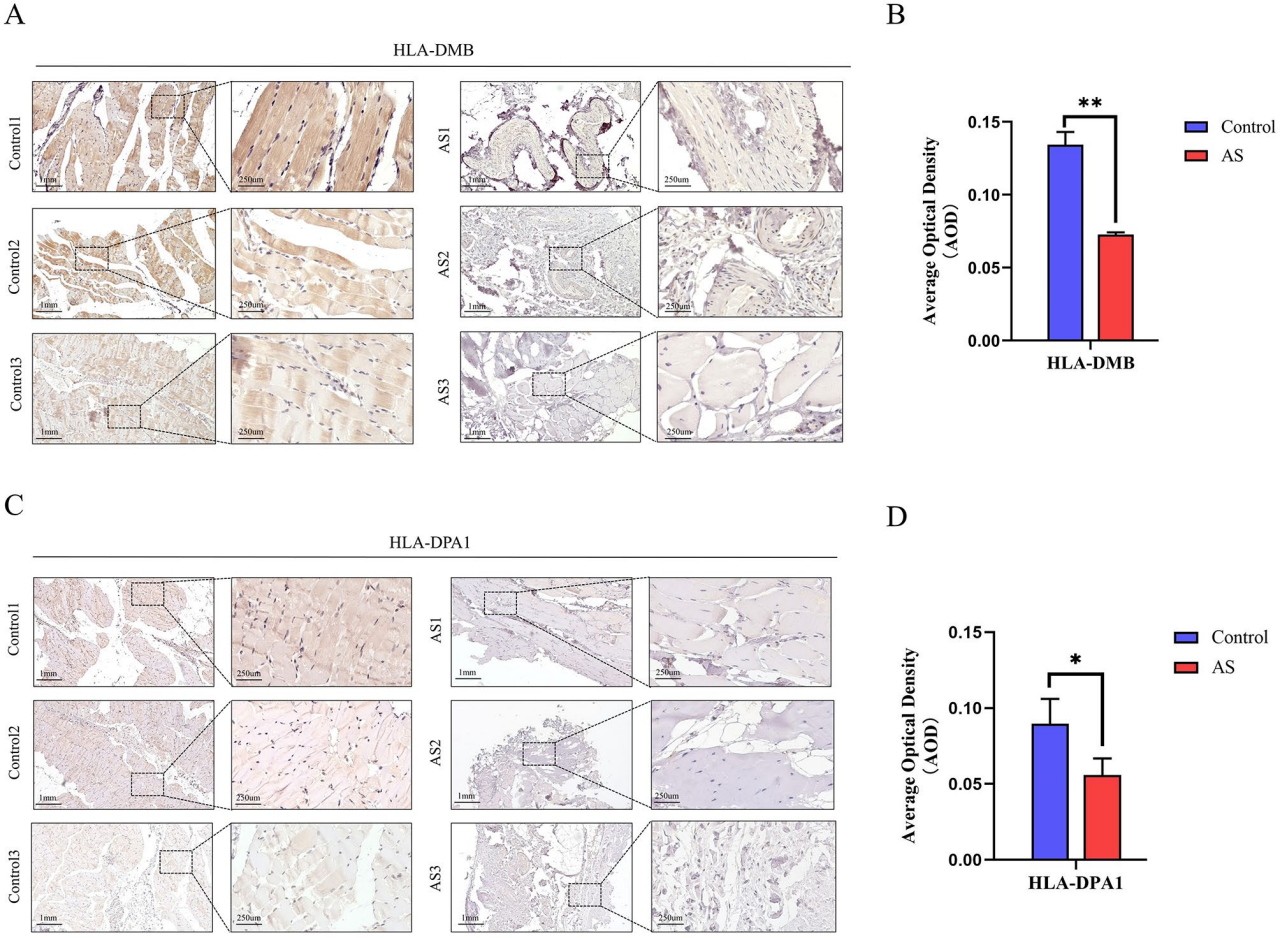
Immunohistochemistry of key immune checkpoint proteins. (**A**) Immunohistochemistry for HLA-DMB in 3 control and 3 ankylosing spondylitis (AS) ligaments samples. Scale bar: 1 mm on the left image, and 250 μm on the right image. (**B**) Quantification and statistical analysis of HLA-DMB immunohistochemistry results. (**C**) Immunohistochemistry for HLA-DPA1 in 3 control and 3 AS ligaments samples. Scale bar: 1 mm on the left image, and 250 μm on the right image. (**D**) Quantification and statistical analysis of HLA-DPA1 immunohistochemistry results

In the AS group, HLA-DMB had an average Average Optical Density (AOD) of 0.0734, compared to 0.1344 in the control group (Fig. 9A, B). Similarly, HLA-DPA1 showed an average AOD of 0.0560 in the AS group and 0.0930 in the control group (Fig. 9C, D). T-test results revealed significant statistical differences (*p* < 0.05) in both comparisons, indicating a notable downregulation of HLA-DMB and HLA-DPA1 expression in AS patients’ hip ligaments. These findings underscore the dysregulation of immune checkpoint proteins in AS.

## Discussion

Immune checkpoints serve as natural mechanisms regulating immune responses to prevent excessive activation and potential harm to healthy tissues [20]. While immune checkpoint inhibition therapy has transformed cancer immunotherapy [21–23], the regulatory role of immune checkpoint genes (ICGs) in immune-related non-cancerous diseases, such as ankylosing spondylitis (AS), remains unclear. This study delved into AS-associated ICGs at both transcriptomic and proteomic levels, employing pathway analysis, immune cell correlation analysis, and drug sensitivity analysis. The objective was to enhance our understanding of how ICGs influence AS development and offer novel strategies for clinical immunotherapy in AS.

The analysis revealed significant downregulation of HLA-DMB and HLA-DPA1 at both gene and protein levels. Immunohistochemistry further confirmed this downregulation. HLA-DMB and HLA-DPA1 are part of the human leukocyte antigen (HLA) complex, expressed on the surface of immune cells, and play a crucial role in the recognition and communication among immune cells [24]. . The HLA-DPA1 gene codes for the MHC-II alpha chain [25], and the HLA-DMB gene codes for the MHC-II beta chain [26]. These genes contribute to the formation of functional MHC-II complexes, crucial for antigen presentation to CD4 + T cells [27]. Abnormal expression of HLA molecules may disrupt antigen presentation accuracy, potentially affecting CD4 + T cell function. Polymorphisms in the HLA-DMB and HLA-DPA1 genes have been linked to autoimmune diseases like rheumatoid arthritis and psoriasis. However, their connection to AS remains less explored [28, 29]. This study’s KEGG analysis uncovered that HLA-DMB and HLA-DPA1 collectively participate in Th1 and Th2 cell differentiation, antigen processing and presentation, as well as Th17 cell differentiation. Th1, Th2, and Th17 cells, subgroups of CD4 + T cells [30], are involved in inflammation, with Th17 cells contributing to autoimmune responses by producing cytokines like IL-17 and IL-22 [31]. Recent studies have observed higher proportions of Th17 cells and increased serum IL-17 levels in the peripheral blood of individuals with AS. Clinical evidence supports the notion that IL-17 plays a pathogenic role in the inflammation observed at AS sites [32].In this study, GSVA analysis identified activated Th17 cell differentiation and the NF-kappa B signaling pathway in the AS group. The NF-κB signaling pathway is pivotal in AS progression, affecting Th1 and Th17 cell differentiation and activation [33–35]. SPI1 activates NF-κB via TLR5 up-regulation, fostering Th1 and Th17 cell differentiation [33]. Additionally, PCSK9 promotes Th1 and Th17 cell proliferation through NF-κB signaling [34]. CX3CL1 exacerbates inflammation and bone changes in AS by promoting M1-type macrophage polarization and bone resorption cell differentiation via this pathway [36]. NF-κB serves as a central regulator of inflammation and bone lesions in AS, making it a key intervention target. This study suggests that ICGs may influence Th17 cell function through the MHC-II complex and NF-κB signaling pathway. Th17 cells, via IL-17 secretion, amplify proinflammatory cytokine production, exacerbating AS inflammation.

xCELL analysis demonstrated notable differences in immune cell infiltration levels between the AS group and the control group, encompassing CD8 + Tcm, CD8 + Tem, CD8 + naive T-cells, CD4 + memory T-cells, and CD4 + Tcm. Following LASSO and intersection analysis, CD8 + Tcm and CD8 + naive T-cell were selected. These findings indicate that, alongside CD4 + T cells, CD8 + T cells play a crucial role in the progression of AS. In AS patients, abnormal aggregation of HLA-B27 molecules occurs within cells [37]. These aberrant HLA-B27 dimers in the cytoplasm may be recognized by CD8 + T cells, triggering autoimmune reactions and tissue damage, ultimately contributing to AS development [38].

The study pinpointed six DEICGs associated with AS, underscoring the involvement of non-MHC genes like LAG3 and SIRPA in regulating immune cell functions. LAG3 (lymphocyte activation 3), a member of the immunoregulatory molecule family, is primarily expressed on CD4 + and CD8 + T cells [39]. Binding of LAG3 to its ligand triggers a negative signal, inhibiting T cell proliferation, function, and effector activity [40]. This negative regulatory mechanism helps prevent excessive immune responses and autoimmune attacks. In AS patients, the downregulation of LAG3 may disrupt the balance of LAG3-mediated negative regulation, potentially resulting in an immune system attack against self-tissues. SIRPA (signal regulatory protein alpha), also known as CD172a, regulates immune cells by binding to its ligand CD47 [41, 42]. CD47 and SIRPA are expressed on specific dendritic cell (DC) subsets and T cells in rats, mice, and humans [43, 44]. Both in vivo and in vitro studies have shown that disruption of the CD47-SIRPA interaction impairs DC function, particularly in triggering Th1, Th2, Th17, and NKT cell responses [45]. Ongoing research indicates that antagonizing the CD47-SIRPA interaction can alleviate clinical symptoms in arthritis, colitis, and Crohn’s disease, making the CD47-SIRPA axis a potential therapeutic target for these conditions [46–48].

This study may be the first to propose a diagnostic model for diseases by combining immune cells and immune checkpoints. Compared to using genes alone as diagnostic markers, the diagnostic efficacy of the model improved from 0.80 to 0.86 by combining genes with immune cells, and the model was successfully validated through an independent external validation set. This enhancement is expected to contribute to more accurate clinical diagnoses of AS in the future.

The drug sensitivity analysis reveals a robust correlation between the drugs Doramapimod and GSK269962A with HLA-DMB, HLA-DPA1, LAG3, and SIRPA. Doramapimod (also known as BIRB 796) is a mitogen-activated protein kinase (MAPK) inhibitor [49]. Excessive activation of the MAPK signaling pathway may lead to pathological inflammation and tissue damage [50]. As a highly selective p38 MAPK inhibitor, Doramapimod mitigates the production of inflammatory cytokines and excessive activation of inflammatory responses by inhibiting the activity of the p38 MAPK signaling pathway [51, 52]. GSK269962A is a selective inhibitor of ROCK1 (Rho-associated protein kinase 1) [53]. In a mouse model of AS, the inhibition of ROCK1 and the activation of p-Erk1/2 in the MAPK signaling pathway were found to suppress the osteoblast-related classical Wnt/β-catenin pathway, thereby alleviating abnormal ossification in AS [54].

This study has several limitations. Firstly, the analysis relied on peripheral blood sequencing data from public databases, which lacked relevant clinical information, making it challenging to perform a comprehensive analysis based on clinical data. Secondly, while the study identified ICGs associated with AS, further experimental investigations are necessary to elucidate the physiological mechanisms through which these ICGs regulate AS. Lastly, the therapeutic potential of the screened drugs was supported solely by literature evidence, and their clinical significance needs to be validated further through additional experiments.

## Conclusions

This study conducted a preliminary exploration into the role of immune checkpoints and immune cells in the treatment and diagnosis of AS. The findings indicated that immune checkpoint proteins HLA-DMB, HLA-DPA1, and immune checkpoint genes LAG3 and SIRPA regulated Th17 cells and CD8 + T cells, influencing inflammation and autoimmune processes in AS. The immune cell populations associated with immune checkpoints exhibited robust diagnostic performance for AS, with AUC values of 0.98, 0.81, and 0.75 for the three models, respectively. Combining immune cells and immune checkpoint genes in model construction enhanced the diagnostic efficiency of the AS diagnostic model. Drug sensitivity analysis, in conjunction with key immune checkpoints, identified potential immunotherapeutic drugs for AS, such as doramapimod and GSK269962A. In conclusion, immune checkpoints and immune cells contribute to the diagnosis and treatment of AS.

### Electronic supplementary material

Below is the link to the electronic supplementary material.

Supplementary Material 1

Supplementary Material 2

Supplementary Material 3

Supplementary Material 4

Supplementary Material 5

Supplementary Material 6

## Data Availability

Data is provided within the manuscript or supplementary information files.

## Abbreviations

AS: Ankylosing spondylitis
LFQ: Label-Free Quantification
IHC: Immunohistochemistry
NSAIDs: Nonsteroidal anti-inflammatory drugs
ICGs: Immune checkpoint genes
MS1: Mass Spectrometry Level 1
PPI: Protein-Protein Interaction
WGCNA: Weighted Gene Co-expression Network Analysis
MAD: Median Absolute Deviation
KEGG: Kyoto Encyclopedia of Genes and Genome
GO: Gene Ontology
GSVA: Gene Set Variation Analysis
IOBR: Immuno-Oncology Biological Research
LASSO: Least absolute shrinkage and selection operator
IC50: 50% inhibitory concentration
OD: Optical density
IOD: Integral optical density
AOD: Average Optical Density
PPI: Protein-protein interaction
DEPs: Differentially expressed proteins
PB: Peripheral blood
DEGs: Differentially expressed gene
PB-DEGs: Differentially expressed genes in peripheral blood
DEICGs: Differentially expressed immune checkpoint genes
AS-DEGs: AS-related differentially expressed genes
CAMs: Cell adhesion molecules
HLA: Human leukocyte antigen
