# Supplementary material for: Exploration of the combined role of immune checkpoints and immune cells in the diagnosis and treatment of ankylosing spondylitis: a preliminary study immune checkpoints in ankylosing spondylitis
Source: Arthritis Res Ther. 2024 Jun 4;26:115. doi: 10.1186/s13075-024-03341-6 (PMC11149331; doi:10.1186/s13075-024-03341-6)
Supplement: Supplementary file 3 — Supplementary Material 3 [file 13075_2024_3341_MOESM3_ESM.docx]

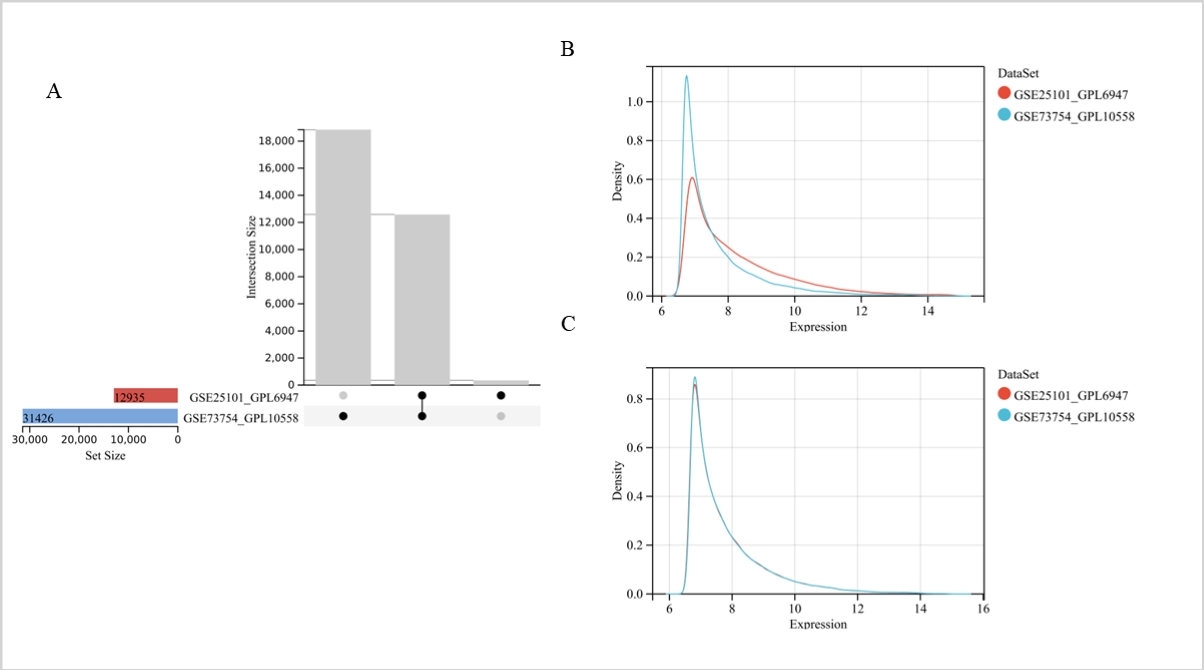


**Supplementary Material 1:** Data preprocessing: (A) Batch effect removal (Set Size: number of genes included in the gene set, bar plot: number of genes shared between the two datasets). (B) Before batch effect removal. (C) After batch effect removal.
