# Supplementary figures and images for: Exploration of the combined role of immune checkpoints and immune cells in the diagnosis and treatment of ankylosing spondylitis: a preliminary study immune checkpoints in ankylosing spondylitis
Source: Arthritis Res Ther. 2024 Jun 4;26:115. doi: 10.1186/s13075-024-03341-6 (PMC11149331; doi:10.1186/s13075-024-03341-6)

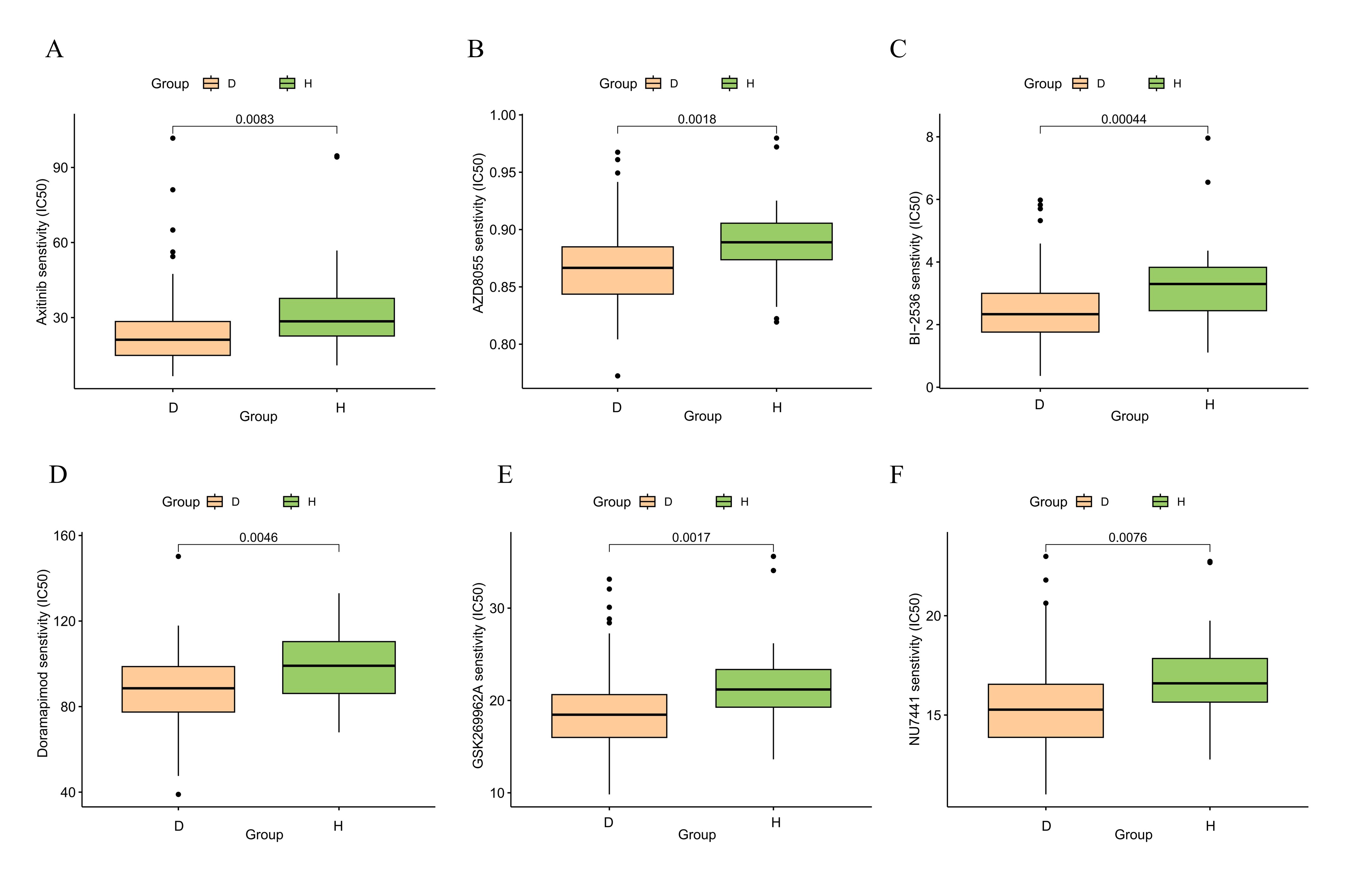

Supplement: Supplementary file 5 — Supplementary Material 5 [file 13075_2024_3341_MOESM5_ESM.jpg]

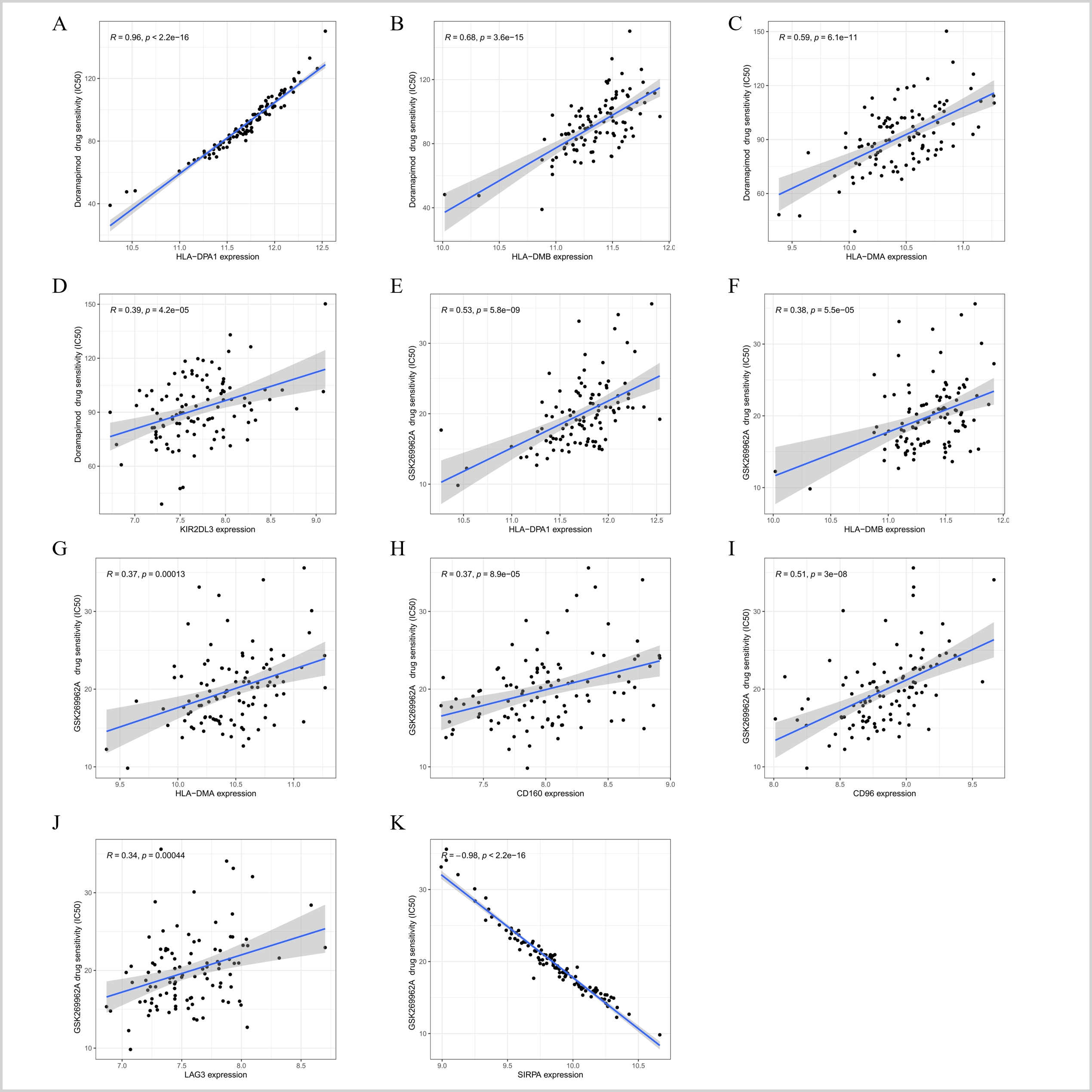

Supplement: Supplementary file 6 — Supplementary Material 6 [file 13075_2024_3341_MOESM6_ESM.jpg]
